# VRAC channel inhibition as a novel strategy for the treatment of ischemia-reperfusion injury

**DOI:** 10.3389/fcell.2024.1524723

**Published:** 2024-12-23

**Authors:** Yuhan Gao, Lu Li, Yuejun Zhang, Yanlong Chu, Guang Han

**Affiliations:** Department of Anesthesiology, Shengjing Hospital of China Medical University, Shenyang, China

**Keywords:** ischemia-reperfusion injury, volume-regulated anion channel (VRAC), LRRC8A, Neuroprotection, therapeutic strategy

## Abstract

Ischemia-reperfusion injury is a serious clinical pathology involving multiple organs such as the heart and brain. The injury results from oxidative stress, inflammatory response and cell death triggered by restoring tissue blood flow after ischemia, leading to severe cell and tissue damage. In recent years, the volume-regulated anion channel (VRAC) has gained attention as an important membrane protein complex. VRAC plays a dual role in ischemia-reperfusion injury: on the one hand, activated VRAC promotes the release of intracellular chloride and glutamate, exacerbating cellular swelling and excitotoxicity, and on the other hand, the regulatory effect of VRAC may also provide protection to cardiomyocytes. This article reviews the pathophysiological mechanisms of ischemia-reperfusion injury, existing therapeutic strategies and their limitations, focuses on the molecular structure of VRAC, its activation mechanism, and its role in ischemia-reperfusion injury, and concludes with a discussion of the potential of targeted inhibition of VRAC as an emerging therapeutic strategy and the challenges it faces. A deeper understanding of the role of VRAC in ischemia-reperfusion injury is expected to provide new therapeutic ideas to improve patient prognosis.

## 1 Introduction

Ischemia-reperfusion injury (IRI) is an important clinical problem that occurs in multiple organs including the heart, brain, liver, kidneys, and intestines, and is a common consequence of acute events such as stroke and myocardial infarction, affecting millions of people worldwide (Roth et al., 2020). Ischemia-reperfusion injury is caused by the restoration of tissue blood flow after an ischemic event, and this restoration triggers oxidative stress, inflammation, and cell death, leading to a cascading series of cellular and molecular events that exacerbate the initial damage caused by ischemia (Pickell et al., 2020). Studies have shown that in myocardial infarction, almost 50% of the final infarct size is attributed to reperfusion injury (Yellon and Hausenloy, 2007), therefore, finding effective therapeutic strategies to mitigate reperfusion injury is important to improve prognosis.

In ischemia-reperfusion injury, multiple ion channels are involved and play important roles. These include a widely expressed ion channel, the volume-regulated anion channel (VRAC) (Nilius et al., 1997). In recent years, with breakthroughs in the study of its structure, VRAC has begun to receive more and more attention (Hou et al., 2022), and it has been suggested that VRAC may play an important role in promoting the damage caused by ischemia-reperfusion to cells and tissues (Okada et al., 2009), and thus inhibition of VRAC channels is being investigated as a novel therapeutic strategy for ischemia-reperfusion injury. This promising therapeutic strategy is described in this review.

## 2 Pathophysiological mechanisms of ischemia-reperfusion injury

**FIGURE 1:**
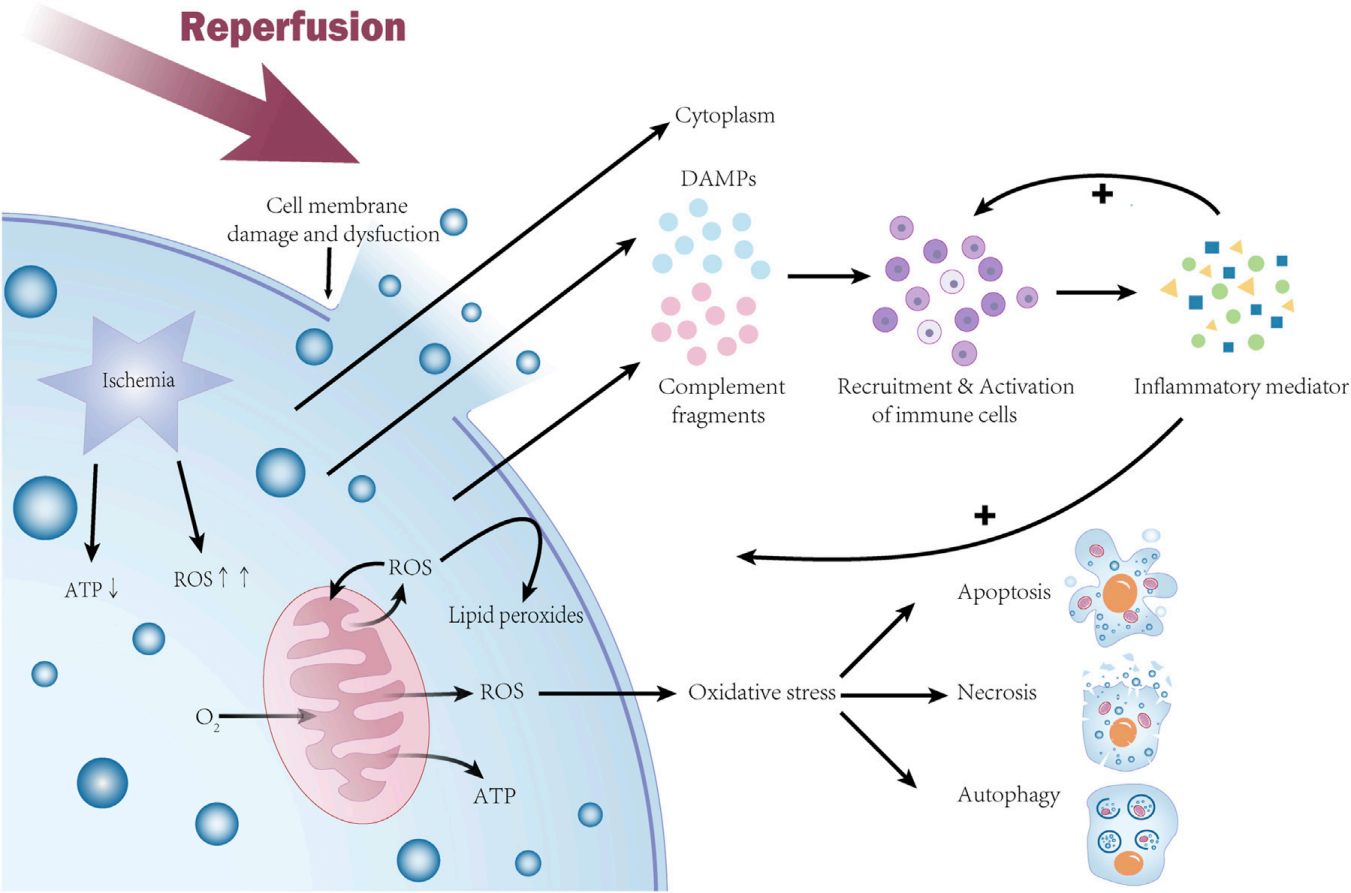
Molecular mechanisms of ischemia-reperfusion injury. During the ischemic phase, cellular ATP production is reduced, mitochondria are dysfunctional and generate excess reactive oxygen species (ROS), leading to oxidative stress that damages cellular structures and initiates the cell death pathway. During the subsequent reperfusion phase, rapid depletion of oxygen and nutrients exacerbates mitochondrial dysfunction and causes a further burst of ROS, activating the cell death program. ROS oxidizes polyunsaturated fatty acids in the cell membrane to produce lipid peroxides, leading to cell membrane damage and loss of function. The inflammatory response exacerbates the damage during the reperfusion phase with the release of damage-associated molecular patterns (DAMPs) and activation of the complement system, causing recruitment and activation of immune cells. Activated inflammatory cells release large amounts of inflammatory mediators that further promote the inflammatory response. Ultimately, these events lead to cell death through multiple mechanisms, including apoptosis, necrosis, and autophagy, causing severe damage to the organism.

Ischemia-reperfusion injury involves a complex series of biological processes (Figure 1), including an initial ischemic phase and a subsequent reperfusion phase, which lead to cell and tissue damage.

### 2.1 Ischemic phase

This initial phase is characterized by oxygen and nutrient deprivation, leading to reduced ATP production and metabolic dysfunction. Mitochondrial dysfunction triggers excessive reactive oxygen species (ROS) generation (Chen et al., 2021; Grass et al., 2022). The accumulation of ROS leads to oxidative stress, damaging cellular structures and initiating cell death pathways. The mitochondrial membrane potential becomes hyperpolarized, further enhancing ROS generation and leading to mitochondrial dysfunction (Grass et al., 2022).

### 2.2 Reperfusion phase

The reperfusion phase is a critical period of ischemia-reperfusion injury, and its negative effects (and also the main characteristics of ischemia-reperfusion injury) are mainly reflected in the following aspects: oxidative stress, inflammatory response, and cell death. Oxygen and nutrients accumulated during ischemia are rapidly depleted during reperfusion, leading to mitochondrial electron transport chain dysfunction and ROS burst (Wang et al., 2011), activating apoptotic and necrotic programs (Zhou et al., 2018). Meanwhile, ROS oxidizes polyunsaturated fatty acids in the cell membrane to produce lipid peroxides, leading to cell membrane damage and loss of function (George et al., 2024). Inflammatory response during the reperfusion phase is also an important cause of increased damage. Prolonged ischemia is associated with increased production of damage-associated molecular patterns (DAMP) released into the surrounding tissues upon cell death. These molecular patterns can increase pro-inflammatory signaling cascades in peripheral cells and activate the complement system, which in turn causes recruitment and activation of immune cells (Kaltenmeier et al., 2022), and activated inflammatory cells release a large number of inflammatory mediators, such as tumor necrosis factor-alpha (TNF-α) and interleukin 6 (IL-6), which further contribute to inflammatory responses (Zhou et al., 2018). In addition, increased vascular permeability, excessive platelet aggregation and release of derived mediators during ischemia-reperfusion injury are alterations that lead to abnormalities in the coagulation process and exacerbate the inflammatory response (Eltzschig and Eckle, 2011). The inflammatory response also leads to a cytokine cascade, the consequences of which include further generation of reactive oxygen species (ROS), tissue hypoxia, and cell death (George et al., 2024). In conclusion, ischemia-reperfusion injury causes cell death through multiple mechanisms involving apoptosis, necrosis, and autophagy (Hotchkiss et al., 2009), resulting in severe damage to the organism, and an understanding of these mechanisms is essential for the development of therapeutic strategies to mitigate ischemia-reperfusion injury-induced tissue damage.

## 3 Treatment and limitations of ischemia-reperfusion injury

Because of its complex pathophysiological mechanisms, current treatments for ischemia-reperfusion injury are limited in effectiveness and scope, highlighting the urgent need for new therapies.

There are no specific treatments for ischemia-reperfusion injury, but several strategies have been widely used to manage the disease and mitigate its effects. These strategies focus primarily on restoring blood flow and preventing further tissue damage; however, these approaches do not adequately address the underlying pathophysiological mechanisms of IRI, which involve inflammatory and immune responses that exacerbate tissue damage (Sánchez-Hernández et al., 2020). For example, in the treatment of acute myocardial infarction (MI), the primary goal is to restore coronary blood flow, which is achieved as quickly as possible with thrombolytic therapy and/or angioplasty (Eltzschig and Eckle, 2011). Although reperfusion is beneficial in reducing the morbidity and mortality associated with MI, the process may also trigger an inflammatory response that further extends the damage caused by the initial ischemia (Eltzschig and Eckle, 2011). Therefore, more therapeutic strategies are being explored.

One such strategy is the use of drugs that target specific pathways involved in ischemia-reperfusion injury. For example, antioxidants can scavenge ROS, reduce oxidative stress and protect ischemic organs (Rodrigo et al., 2022). Moreover, inhibition of the complement system, which attenuates the immune-inflammatory response triggered by reperfusion, is currently being investigated as a means of reducing IRI (Granger et al., 2003). However, in animal experiments using this approach, some studies have reported improved survival but no reduction in infarct size (de Zwaan et al., 2002; Verrier et al., 2004). This suggests that although complement inhibition may modulate some aspects of ischemia-reperfusion injury, it may not be able to completely eliminate the injury process.

Ischemic preconditioning (IPC) and postconditioning (IPostC) are also two strategies for treating ischemia-reperfusion injury. They protect organs and reduce infarct size by a short ischemia/reperfusion cycle before or after reperfusion of the ischemic area, respectively (Kumar et al., 2021). Ischemic preconditioning removes accumulated catabolic metabolites and reduces ATP depletion, protecting organs from damage caused by subsequent prolonged ischemia (Murry et al., 1991), but needs to be performed before the ischemic event, which is difficult to predict in clinical practice (Lim and Hausenloy, 2012). For postconditioning, which is theoretically more feasible in the clinic, more in-depth studies are still needed (Kumar et al., 2021).

Mesenchymal stem cells (MSCs) can be used to treat ischemia-reperfusion injury by secreting multiple trophic factors to inhibit inflammatory signals and oxidative stress, and clinical trials applying MSCs to treat ischemia-reperfusion injury are currently underway (Arakawa et al., 2023). However, the safety of MSC therapy needs to be emphasized. IPSC-derived MSCs (iMSCs) have an unlimited self-renewal capacity and therefore have tumorigenic potential (Liu et al., 2013). In addition, MSCs expressing tissue factor (TF/CD142) have the risk of causing thromboembolism and thus have limitations in clinical application (Arakawa et al., 2023).

The role of neutrophils in IRI has also become a focus of therapeutic intervention in IRI. The use of CD11/CD18 integrin-blocking antibodies inhibits neutrophil aggregation in the infarcted area. Although animal studies have shown promise, clinical trials have not demonstrated a significant reduction in infarct size or improvement in clinical outcomes (Faxon et al., 2002).

Statins such as Rosuvastatin inhibit the inflammatory response during ischemia-reperfusion by mediating the accumulation of immunosuppressive regulatory T cells (Tregs) (Burne-Taney et al., 2006; Arias-Diaz et al., 2009) in a mouse model, which in turn limits IRI (Ke et al., 2013). This suggests that statins may have a role that can be used in IRI therapy.

In conclusion, IRI therapeutic strategies need to have a multifaceted role, and exploring new therapeutic strategies requires a nuanced understanding of the complex interactions between inflammatory mediators, immune cells, and tissue repair mechanisms. Current therapeutic strategies, while showing promise in preclinical and some clinical settings, have yet to yield clear and universally effective approaches to IRI management. Recently, it has been found that the volume-regulated anion channel (VRAC) also plays an important role in ischemia-reperfusion injury, and that inhibition of VRAC can attenuate the damage caused by ischemia-reperfusion, and research on VRAC and its inhibitors is expected to drive new advances in the treatment of ischemia-reperfusion injury. The following section describes this in detail.

## 4 VRAC channels and their molecular components

### 4.1 Composition and structure of VRAC

The volume-regulated anion channel (VRAC) is a key membrane protein complex that plays an important role in cell volume regulation and various physiological processes. Its molecular identity remained an enigma for nearly 3 decades until its essential subunit leucine-rich repeat-containing protein 8A (LRRC8A, also known as SWELL1) was discovered (Qiu et al., 2014). VRAC channels are formed by the assembly of LRRC8 family, specifically LRRC8A and its four homologous proteins LRRC8B, LRRC8C, LRRC8D and LRRC8E (Voss et al., 2014). These proteins form heterotrimeric multimeric channels (Syeda et al., 2016), and the composition of the subunits determines the properties and substrate selectivity of the channels, giving the constituent VRACs different characteristics, including their ionic selectivity and permeability to organic osmotic pressure regulators (Osei-Owusu et al., 2018).

The LRRC8 protein consists of two parts: four transmembrane helices (TM) at the N-terminal end and seventeen leucine-rich repeat sequences (LRR) at the C-terminal end (Abascal and Zardoya, 2012). The LRR structural domain is intracytoplasmic and is thought to be involved in protein-protein interactions. The TM region displays weak, but significant, sequence similarity to gap junction proteins pannexins, suggesting a possible evolutionary relationship (Osei-Owusu et al., 2018). Functional studies and mutational analyses have established that the N-terminal TM region plays an important role in the structure and function of VRAC, such as the threonine (T44) at position 44 in SWELL1, where mutation of this residue significantly alters the anion permeability ratio (Qiu et al., 2014; Syeda et al., 2016).

### 4.2 Physiological mechanism

VRAC can be activated by a variety of stimuli, and its main activation mechanism is cell swelling, extracellular hypo-osmotic state or intracellular hyperosmotic state leading to an osmotic pressure gradient that triggers the opening of VRAC channels, mediating the transmembrane transport of a variety of anions and the formation of a kind of anionic currents to restore cell volume (Hoffmann et al., 2009; Jentsch, 2016). VRAC is therefore thought to play an important role in the cellular regulated volume reduction (RVD) role (Formaggio et al., 2019), and it has been shown that when LRRC8 gene expression is inhibited, the intensity of the VRAC current decreases and the ability to regulate cell volume is significantly reduced (Formaggio et al., 2019). VRAC can also be activated by a decrease in intracellular ionic strength, and it has been suggested that this is the initial trigger for VRAC activation (Osei-Owusu et al., 2018), rather than an increase in cell volume *per se*. In addition, the mechanism of VRAC activation may also involve a variety of intra- and extracellular signaling molecules. For example, the inflammatory mediator sphingosine-1-phosphate (S1P) can activate VRAC in microglia, and S1P-induced VRAC activation can lead to the release of ATP, which may further affect microglial and neuronal activity and cause neuropathic pain (Chu et al., 2023). Other activation mechanisms such as apoptosis inducers like staurosporine and cisplatin (Maeno et al., 2000; Shimizu et al., 2004), and G protein-coupled receptor (GPCR) (Liu et al., 2009; Takano et al., 2005) activation have also been reported.

### 4.3 Physiological effects

The physiological mechanisms of VRAC are mainly involved in cell volume regulation and regulation of multiple physiological processes. Its most prominent role is mediating anion release and regulating cell volume as mentioned above, in addition to several other aspects, such as involvement in cell proliferation (Shen et al., 2000), apoptosis (Konishi et al., 2019), necrosis (Liu and Stauber, 2019), and migration (Liu and Stauber, 2019); In terms of metabolism, VRAC has been shown to be critical for glucose-stimulated insulin secretion in pancreatic islet β-cells. The channel contributes to membrane depolarization, causing calcium inward flow and subsequent insulin release (Best et al., 2010); Immunologically, it plays a role in the activation of the NLRP3 inflammasome, which is a key component of the innate immune response (Compan et al., 2012), in addition, VRAC contributes to T-cell activation and B-cell development, as evidenced by the phenotype of Swell1 knockout mice, which show deficits in T-cell development and B-cell function (Kumar et al., 2014); VRAC also exerts an effect on the nervous system, it is involved in the release of excitatory neurotransmitters such as glutamate (Mongin, 2016). This process may have a greater impact in conditions such as ischemic stroke, where glutamate release mediated by VRAC activated by cerebral ischemia causes damage to neurons through excitotoxicity (Zhou et al., 2020).

As the understanding of the physiological role of VRAC deepens, some emerging areas are gradually gaining attention from researchers. Since VRAC is permeable to certain apoptosis inducers such as cisplatin, it contributes to the cellular uptake of anticancer drugs (Osei-Owusu et al., 2018). Studies have shown that downregulation of the SWELL1 subunit correlates with cancer cell resistance to cisplatin (Sørensen et al., 2016), suggesting that VRAC may be a promising target for overcoming cancer cell resistance in cancer therapy. Improving cancer therapeutic efficacy by modulating VRAC activity may become a promising study in the future.

Another area that is still unclear but holds great promise is the modulation of neuropathic pain by VRAC. Given that VRAC involves the release of ATP and glutamate (Chu et al., 2023), a key neurotransmitter involved in pain signaling (Fundytus, 2001), the role played by VRAC in pain perception and transmission warrants further study to identify potential therapeutic opportunities.

In conclusion, the physiological role of VRAC encompasses many aspects of the organism’s vital activities such as cell volume regulation, immune responses, metabolic processes, and neurological functions, and an understanding of the biophysical properties and regulatory mechanisms of VRAC is essential for the development of effective therapeutic approaches.

## 5 VRAC’s role in the pathophysiology of ischemia-reperfusion

In the context of ischemia-reperfusion injury, cells are altered by ischemic osmolarity and undergo swelling, activating volume-regulated anion channels (VRAC), which play an important role.

In the CNS, it has been shown that VRAC activity is upregulated in neurons and astrocytes in the brain of stroke mice (Chen et al., 2024), leading to an increase in intracellular chloride ion efflux thereby inducing cytotoxic neuronal swelling and glutamate excitotoxicity effects, which aggravate the brain damage of ischemic stroke through a dual effect (Chen et al., 2024). Reduced neuronal swelling was observed in neuron-specific Swell1 knockout (NEX-cKO) mice, it can therefore be hypothesized that this swelling is dependent on SWELL1 channels (Chen et al., 2024), suggesting that SWELL1 plays an important role in cerebral ischemia-reperfusion injury. Activation of VRAC in astrocytes is thought to release glutamate (Yang et al., 2019), and large amounts of glutamate cause overactivation of NMDA receptors, exacerbating excitotoxicity and neuronal death (Belov Kirdajova et al., 2020).

**FIGURE 2:**
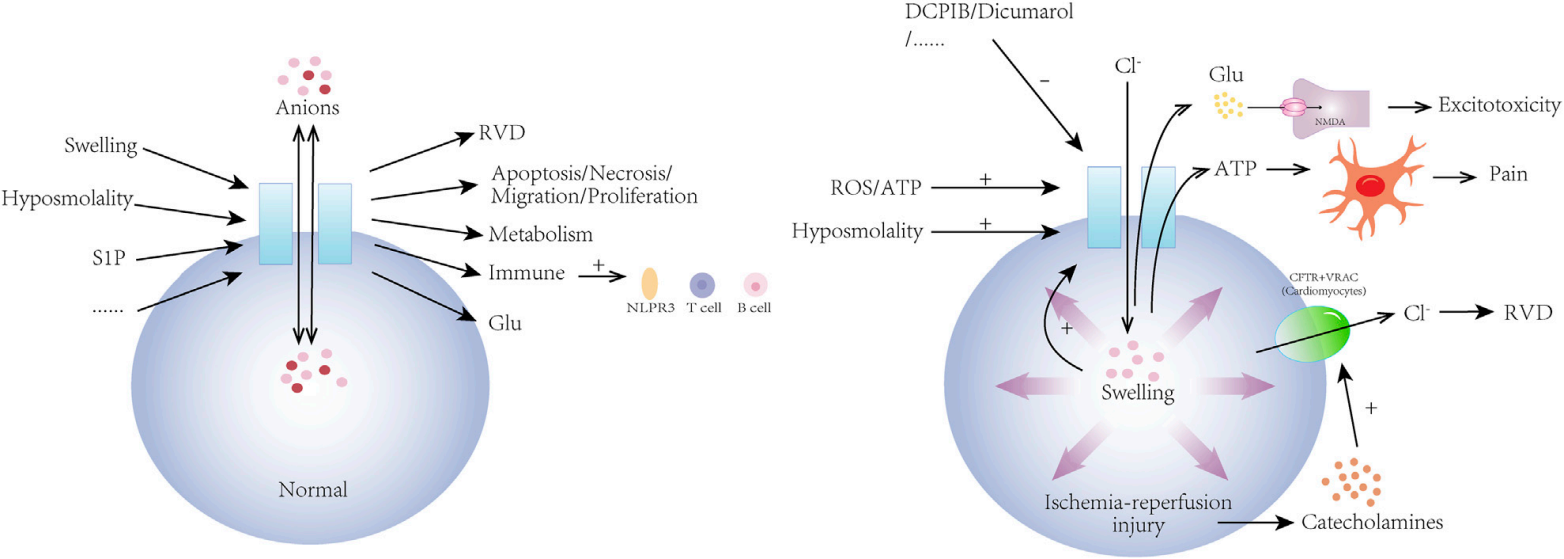
Physiological mechanisms of volume-regulated anion channel (VRAC) and its role in ischemia-reperfusion injury. VRAC channels are activated by a variety of physiological and pathological stimuli including hyposmolality, sphingosine-1-phosphate (S1P), and cellular swelling. They are involved in the regulation of cellular volume (RVD) and a wide range of cellular activities by mediating the transport of a variety of anions across membranes. In addition, VRAC channels are involved in the regulation of metabolism, the immune system, and the nervous system. In ischemia-reperfusion injury, VRAC is activated by hyposmolality, ROS/ATP, and then pathologically releases glutamate and ATP, causing neuronal swelling, glutamatergic excitotoxic effects, and pain. In ischemia-reperfusion injury of cardiomyocytes, VRAC regulates cell volume and function in conjunction with cystic fibrosis transmembrane conductance regulator (CFTR). VRAC activity can be inhibited by drugs such as DCPIB and dicumarol.

VRAC also plays a similar double-edged role in myocardial ischemia-reperfusion injury (Okada et al., 2019). During myocardial ischemia-reperfusion, the heart undergoes deprivation of oxygen and nutrients during ischemia and reperfusion, leading to cellular swelling and activation of VRAC. The protection of cardiomyocytes by VRAC may be related to the cardiac cystic fibrosis transmembrane conductance regulator (cCFTR), a ventricular splice variant of CFTR (Okada et al., 2019). CFTR is a cyclic adenosine monophosphate (cAMP)-dependent chloride channel expressed in the myocardium, whose function involves participation in the transport of chloride ions (Levesque et al., 1992). At the onset of myocardial ischemia-reperfusion injury, catecholamines are released in large quantities (Karwatowska-Kryńska and Beresewicz, 1983), and cCFTR is subjected to β-adrenergic stimulation (Bahinski et al., 1989; Harvey and Hume, 1989; Ehara and Ishihara, 1990; Matsuoka et al., 1990), which participates in the RVD mechanism (Wang et al., 1997; Yamamoto et al., 2001), and acts in conjunction with VRAC, which helps to restore cell volume homeostasis by facilitating the efflux of chloride ions. Although there seems to be no study showing a direct correlation between the roles played by VRAC and CFTR in the RVD process of cardiomyocytes when ischemia-reperfusion injury occurs, during the phase of myocardial ischemia, intracellular ATP depletion prevents the activation of VRAC (Okada et al., 2019), and CFTR may provide myocardial protection in the event that VRAC activity is impaired, complementing the function of VRAC in regulating cardiomyocyte RVD. However, large amounts of ROS (Wang et al., 2005) and ATP (Clemens and Forrester, 1981) are usually produced intracellularly during reperfusion, which may promote the release of ATP and glutamate from VRAC (Okada et al., 2020), further exacerbating the injury and leading to cell death (Figure 2).

In conclusion, VRAC plays an important role in ischemia-reperfusion injury in both the brain and the heart, and its activation leads to cell swelling, dysfunction, and even death, which in turn affects the overall function of the nervous system and the heart. Therefore, specific targeted inhibition of VRAC may be a novel therapeutic approach to attenuate ischemic cardio-cerebral injury and improve the prognosis of patients with ischemic diseases.

## 6 Therapeutic potential of VRAC inhibition

Inhibition of VRAC may help to reduce the damage caused by ischemia-reperfusion, and inhibition of VRAC by genetic and pharmacological methods has emerged as a promising strategy for the treatment of ischemia-reperfusion injury. The following section describes commonly used methods of VRAC inhibition and their effectiveness in animal models and explores the current limitations of these methods.

### 6.1 Methods of VRAC inhibition

Suppression of VRAC by genetic methods is mainly through conditional knockout of the gene encoding LRRC8A, an essential subunit of VRAC. Yang et al.'s experiments resulted in the deletion of the LRRC8A subunit of VRAC by conditional knockout of the GFAP gene in mouse astrocytes and the GFAP - cKO mice exhibited reduced infarct size and significant improvement in neurological prognosis after middle cerebral artery occlusion (MCAO) (Yang et al., 2019). Subsequently, Zhou et al. showed that ischemia increases LRRC8A-dependent VRAC activity in hippocampal neurons, which enhances glutamate release and contributes to ischemic brain injury (Zhou et al., 2020), and that mice with conditional knockout of the LRRC8A gene had significantly lower infarct volume and neurological severity scores than wild-type mice after middle cerebral artery occlusion cerebral infarction (Zhou et al., 2020). Furthermore, these findings emphasize the importance of astrocyte VRAC in the pathogenesis of ischemic stroke and suggest that targeting this pathway may provide neuroprotection.

A variety of drugs have been shown to inhibit VRAC. 4-(2-butyl-6,7-dichloro-2-cyclopentyl-1-on-5-yl) oxalylbutyric acid (DCPIB) is a potent and specific blocker of VRAC, acting on the swell1 subunit (Decher et al., 2001), and in a reversible middle cerebral artery occlusion (rMCAO) model, in-chamber administration of DCPIB resulted in a reduction in mean infarct volume by approximately 75% compared to controls (Zhou et al., 2020); in Qiu et al. DCPIB attenuated excitotoxicity during ischemic events by decreasing glutamate release, thereby reducing neuronal damage (Qiu et al., 2014); Han et al. demonstrated that DCPIB significantly inhibited hypoxia-glucose deprivation (OGD)-induced activation of microglia, with possible mechanisms including direct inhibition of VRAC in microglia and inhibition of the MAPK pathway, a key pathway mediating microglial activation (Han et al., 2014). Activation of microglia plays an important role in the inflammatory response of ischemia-reperfusion injury (Wang et al., 2023). In a recent study, Cao et al. found that DCPIB could promote the conversion of pro-inflammatory M1 microglia into anti-inflammatory M2 microglia through the MAPK signaling pathway, thereby reducing inflammatory responses and oxidative stress at the ischemic site (Cao et al., 2024). All these studies suggest that DCPIB protects neuronal cells from ischemic injury. In a model of ischemia-reperfusion injury in cardiomyocytes, DCPIB was shown to inhibit chloride efflux mediated by VRAC to attenuate autophagy and oxidative stress in cardiomyocytes (Shen et al., 2014; Xia et al., 2016). These findings suggest that inhibition of VRAC activity using DCPIB may be a promising therapeutic strategy to attenuate ischemia-reperfusion injury.

Dicumarol, another VRAC inhibitor, administered intracerebrally effectively blocked VRAC-mediated anionic currents activated by ischemic stroke in mice (Formaggio et al., 2019), and also inhibited cellular release of ATP (Formaggio et al., 2019) as well as pathologic release of glutamate (Chen et al., 2024), attenuated neuronal swelling and cell death, and provided neuroprotection after ischemic stroke, which is a promising therapeutic strategy for attenuating potential therapeutic agent for ischemic stroke-induced brain damage. In addition, Dicumarol is an FDA-approved drug (Chu et al., 2023). It has been used as an effective anticoagulant in clinical practice for the treatment of deep vein thrombosis and atrial fibrillation and is also recommended by current guidelines for the prevention of ischemic stroke in patients with high-risk atrial fibrillation (Kleindorfer et al., 2021).

In addition to DCPIB and dicumarol, several other VRAC inhibitors have shown potential in the treatment of ischemia-reperfusion injury.

Tamoxifen, a non-selective VRAC inhibitor, has been shown to inhibit injury after cerebral ischemia (Kimelberg et al., 2000), as well as inhibit nNOS (Osuka et al., 2001), scavenge oxygen free radicals (Zhang et al., 2007), and have some neuroprotective ability.

IAA-94 is also a VRAC blocker, and whole-cell membrane slice technology has demonstrated that it can inhibit VRAC currents and reduce glutamate-induced neuronal necrosis (Inoue et al., 2005).

4,4′-diisothiocyanatostilbene-2,2′-disulfonic acid disodium salt hydrate (DIDS), a stilbene derivative chloride channel blocker, effectively eliminates chloride inward flow and neuronal swelling induced by intracellular sodium accumulation during ischemia-reperfusion injury (Chen et al., 2024).

Other drugs such as the carboxylate analog (NPPB), and phloretin have also been shown to inhibit VRAC (Inoue et al., 2005), but their roles in ischemia-reperfusion injury need to be further investigated.

When exploring the clinical potential of VRAC inhibitors, we must consider their efficacy, safety, and pharmacokinetic properties in the treatment of ischemia-reperfusion injury. VRAC inhibitors, such as DCPIB and Dicumarol, have been shown to have a certain ability to attenuate ischemia-reperfusion injury in animal models. However, there are certain limitations in the clinical application of these drugs, which should be addressed in order to fully utilize the therapeutic potential of VRAC inhibitors.

### 6.2 Limitations of currently used VRAC inhibitors

Currently, VRAC inhibitors are not yet used in the clinical treatment of ischemia-reperfusion injury. Because of the limitations of each of these approaches, it is important to optimize the pharmacokinetic and pharmacodynamic properties of these inhibitors and to explore their efficacy in preclinical and clinical settings.

#### 6.2.1 Poor selectivity

Most of the existing VRAC inhibitors have poor selectivity (Figueroa and Denton, 2022). Although DCPIB is a potent VRAC inhibitor, it is somewhat toxic and poorly selective to cells, and can have excitatory or inhibitory effects on other ion channels and transporter proteins (Bowens et al., 2013; Lv et al., 2019; Deng et al., 2016; Ye et al., 2009), and thus may have potential side effects. Exploring more selective VRAC blockers is essential to minimize side effects and maximize therapeutic efficacy.

#### 6.2.2 Poor penetration of the blood-brain barrier (BBB)

Many VRAC blockers do not effectively cross the blood-brain barrier, limiting their clinical use in the treatment of cerebral ischemia. The protective effect of DCPIB intracerebral injection on neurons did not show the same therapeutic effect when administered intravenously at the same dose, suggesting that DCPIB does not effectively penetrate the blood-brain barrier when administered systemically (Zhang et al., 2008). The same problem exists with dicoumarol as a therapeutic agent for ischemia-reperfusion injury this problem, and in animal experiments, the experimenter generally delivers the drug directly to the ischemic region in the brain. This might be a potential solution that can be referred to for clinical application (Nozohouri et al., 2020; Mehta et al., 2017). Further studies are necessary to optimize its delivery through the blood-brain barrier in order to achieve its full therapeutic potential.

#### 6.2.3 Inhibits RVD

VRAC plays a double-edged role in myocardial ischemia-reperfusion injury, i.e., it can both cause myocardial injury and protect cardiomyocytes by regulating cell volume, and its protective effects are inhibited by VRAC inhibitors. For example, it has been shown that IAA-94 inhibits the protective effects of ischemic preconditioning (Batthish et al., 2002; Diaz et al., 1999) and inhibits VRAC-mediated RVD responses (Batthish et al., 2002).

VRAC inhibitors provide a new approach for the treatment of ischemia-reperfusion injury, and it is a promising therapeutic strategy. However, to realize this clinical potential, future research needs to focus on optimizing the pharmacokinetics and pharmacodynamic characteristics of these inhibitors, addressing the challenges related to poor selectivity, poor permeability through the blood-brain barrier, and difficulty in maintaining a balance between protection and damage. Furthermore, it is necessary to explore the efficacy of these inhibitors in preclinical studies to enable them to be used more effectively.

## 7 Conclusion

Ischemia-reperfusion causes severe damage to the organism, and due to the complexity of its pathophysiological mechanisms, it has not been found to find a very effective treatment. VRAC are membrane proteins that are widely expressed in mammalian cells, are activated mainly by changes in cell volume and osmolarity, are involved in a series of physiological processes in the body, and play an important role in ischemia-reperfusion injury. VRAC is activated in the context of ischemia-reperfusion injury and mediates an increase in chloride inward flow and glutamate release in the central nervous system, causing cytotoxic neuronal swelling and glutamatergic excitotoxic effects that exacerbate brain damage in ischemic stroke. In myocardial ischemia-reperfusion injury, VRAC also plays a double-edged role, either causing myocardial injury or exerting a protective effect on cardiomyocytes by regulating cell volume.

Targeted inhibition of VRAC may be a novel therapeutic approach to attenuate ischemia-reperfusion injury and improve the prognosis of patients with ischemic disease. For example, drugs such as DCPIB and dicumarol have been shown to inhibit VRAC, attenuate cell swelling and death, and provide a protective effect for ischemic tissue cells. However, these inhibitors still face challenges in clinical application, including poor selectivity, inadequate blood-brain barrier penetration, and possible inhibition of the protective effects of VRAC in cardiomyocytes.

VRAC inhibitors offer a promising new avenue for the treatment of ischemia-reperfusion injury. Future studies need to focus on optimizing the pharmacokinetic and pharmacodynamic properties of VRAC inhibitors, improving their selectivity and blood-brain barrier penetration, and further exploring their efficacy in preclinical and clinical settings. A deeper understanding of the mechanism of action of VRAC in different organs should also be pursued, which will help develop safer and more effective therapeutic strategies and contribute to the research on the treatment of ischemia-reperfusion injury.
